# Enhanced Thermal Conductivity of Polyamide-Based Nanocomposites Containing Graphene Oxide Sheets Decorated with Compatible Polymer Brushes

**DOI:** 10.3390/ma14040751

**Published:** 2021-02-05

**Authors:** Łukasz Łątka, Kamil Goc, Czesław Kapusta, Szczepan Zapotoczny

**Affiliations:** 1 Faculty of Chemistry, Jagiellonian University, Gronostajowa 2, 30-387 Krakow, Poland; lat.lukasz@gmail.com; 2 Research and Development Center, Azoty Group S.A., Kwiatkowskiego 8, 33-101 Tarnow, Poland; 3 Faculty of Physics and Applied Computer Science, AGH University of Science and Technology, Mickiewicza Av. 30, 30-059 Krakow, Poland; kamil.goc@fis.agh.edu.pl (K.G.); kapusta@agh.edu.pl (C.K.)

**Keywords:** nanocomposites, polymer brushes, graphene oxide, thermal conductivity

## Abstract

Polyamide-based nanocomposites containing graphene platelets decorated with poly(acrylamide) brushes were prepared and characterized. The brushes were grafted from the surface of graphene oxide (GO), a thermally conductive additive, using atom transfer radical polymerization, which led to the formation of the platelets coated with covalently tethered polymer layers (GO_PAAM), accounting for ca. 31% of the total mass. Polyamide-6 (PA6) nanocomposites containing 1% of GO_PAAM were formed by extrusion followed by injection molding. The thermal conductivity of the nanocomposite was 54% higher than that of PA6 even for such a low content of GO. The result was assigned to strong interfacial interactions between the brushes and PA6 matrix related to hydrogen bonding. Control nanocomposites containing similarly prepared GO decorated with other polymer brushes that are not able to form hydrogen bonds with PA6 revealed no enhancement of the conductivity. Importantly, the nanocomposite containing GO_PAAM also demonstrated larger tensile strength without deteriorating the elongation at break value, which was significantly decreased for the other coated platelets. The proposed approach enhances the interfacial interactions thanks to the covalent tethering of dense polymer brushes on 2D fillers and may be used to improve thermal properties of other polymer-based nanocomposites with simultaneous enhancement of their mechanical properties.

## 1. Introduction

The discovery and isolation of single graphene flakes have initiated tremendous interest in this material for both fundamental studies and industrial applications. Research and development studies on graphene-based materials have been carried out on a large scale, but the number of commercial applications is rather limited [1,2]. Graphene materials, due to their high strength as well as electrical and thermal conductivity, are natural candidates for plastics additives. The main issues that need to be addressed to introduce graphene-based material into the market in the form of plastic nanoadditives are the high production costs of graphene and problems with its homogeneous dispersion in the polymer matrix [3]. The interfacial properties between the polymer matrix and the nanoadditive significantly influence the final properties of the nanocomposite and homogeneous distribution of fillers [4,5].

Polyamide 6 (PA6) is an engineering thermoplastic material commonly used as a replacement of metals in various applications due to its excellent performance-cost ratio. Based on the market analysis, nearly 46.3% of the global market of thermally conductive plastics is based on polyamides [6]. PA6 nanocomposite with enhanced thermal conductivity (TC) is a product expected by the market. Considering that polymers exhibit low TC (commonly below 1.0 W/(m K)), making high TC polymer-based materials requires the introduction of high TC filler particles into the matrix [7,8]. Heat transport is realized by transporting phonons and/or electrons. Generally, the fillers with only a phonon heat transfer mechanism have lower TC than those with both phonons and electrons involved in the transfer. Electrons are generally more resistant to scattering and move faster than phonons in highly conductive materials. Thus, even some ceramic materials, such as aluminum or boron nitrides, exhibit high TC; metallic- and carbon-based fillers show typically much higher TC, mainly due to the presence of free electrons. It is worth emphasizing that nanocomposites with the carbon-based fillers may be considered as easy-to-recycle and cost-effective ones, especially if properly modified graphene oxide and not expensive graphene is used.

Polymer nanocomposites containing graphene-based fillers have been attracting a growing interest due to the possibility of achieving materials with significantly improved gas barrier properties, electrical conductivity, thermal conductivity or mechanical strength [9,10,11,12,13]. The most common methods of preparing polymer nanocomposites containing graphene-based fillers are in situ polymerization, melt compounding and solvent blending [2]. There are recent works that focused on achieving high thermal conductivity of graphene-based PA6 composites. Ding et al. [14] presented an increase in TC by 112% for PA6-based nanocomposite containing 10% of reduced graphene oxide (GO) compared to the pristine PA6. The other studies reported an increase of more than 400% in the flexural modulus of PA6 after the addition of 20% graphene nanoplatelets, but lower flexural strength values were found than for nanoclay composites [15]. This suggested that the interface conditions of graphene and PA6 were not optimized, resulting in low strain detaching of the nanofillers. Other approaches utilized modified graphene-based fillers, such as reduced GO with titanate coupling agent [16], graphene stabilized with GO [17] or 3D nanofiller composed of silicon carbide nanowires and graphene sheets [18] to reach very high TC values of the PA6-based nanocomposites but without presentation of their mechanical characterization.

There are some reports on using controlled polymerizations, such as atom transfer radical polymerization (ATRP) for surface derivatization of graphene. Gonalves et al. [19] showed the GO-based reinforcement filler decorated with poly(methyl methacrylate) chains. Firstly, 2-bromo-2-methylpropionyl bromide (BMPB) initiators were immobilized on the surface of GO via (1) esterification of the carboxylic groups of GO by ethylene glycol, followed by (2) attachment of BMPB to such formed hydroxyl groups on the surface of GO. Another report showed the possibility to control chain length and grafting density of polystyrene brushes obtained using ATRP on graphene nanosheets [20]. Liu et al. [21] reported the preparation of GO decorated with poly(amidoxime) brushes by grafting poly(acrylonitrile) chains via surface-initiated ATRP (SI-ATRP) and subsequent formation of amidoxime groups from the nitrile ones. Zygo et al. [22] showed modifications of the GO surfaces by selected poly(methacrylates) at various grafting densities. Nevertheless, to the best of our knowledge, there are no reports demonstrating enhanced mechanical and thermal properties of polyamide-based nanocomposites containing GO sheets decorated with densely grafted polymer brushes and the influence of the type of brushes on such properties.

Owing to the small mixing entropy of macromolecules and nanofillers, rather favorable enthalpic interactions can promote the dispersion of nanofillers leading to the formation of a stable nanocomposite [23]. Especially, the nanofiller/polymer interface structure and the interfacial interactions have a strong impact on the nanocomposite properties. Thus, the formation of hydrogen bonds between the matrix macromolecules and the compatible chains decorating the surface of nanofillers in nanocomposites can not only improve the mechanical properties of the matrix but also thermal conductivity by reducing the phonon scattering. This is especially true for covalently surface-tethered chains as in the polymer brush systems. Molecular dynamics calculations performed for nanocomposite systems containing decorated graphene sheets in the polyamide [8] or poly(lactic acid) [24] matrix indicated that the interfacial TC in the direction perpendicular to the graphene plane is enhanced by the grafted chains and is proportional to the grafting density. The model studies indicated also that incorporating hydrogen bonding between the grafted and matrix chains can effectively improve wetting of the grafted layer, leading to better particle dispersion and integration in nanocomposites [25]. Interpenetration of matrix chains into the grafted layer is possible, if grafted chains extend toward the matrix chains to form donor-acceptor contacts. As a result, the thermomechanical properties of nanocomposites with H-bonding interactions between graft and matrix polymers may also be improved compared to the systems without such interactions.

The presented work reports the synthesis and characterization of GO decorated with poly(acrylamide) polymer brushes (GO_PAAM) as well as the preparation and testing of PA6 composites (PA6/GO_PAAM) with only 1% addition of such modified 2D filler. Importantly, the PA6 matrix macromolecules can form hydrogen bonds with the surface-tethered poly(acrylamide) (PAAM) chains. For a comparison, the composites containing the same content of the nanofillers decorated with other polymer brushes, which cannot form hydrogen bonds with PA6 chains, were also prepared, and their mechanical properties and thermal conductivities were studied.

## 2. Materials and Methods

### 2.1. Materials

Graphene oxide (GO, c.a. 40% content of carbon) was purchased from the Institute of Electronic Materials and Technology (Warszawa, Poland). Cryogenic milled polyamide-6 (PA6) was kindly supplied by Azoty Group S.A. (Tarnów, Poland). Methanol (p.a.) and tetrahydrofuran (THF, p.a.) were bought from Chempur (Piekary Slaskie, Poland) and used as received. Other chemicals, ethanol (p.a.), 3-aminopropyl-trimethoxysilane (APTES), triethylamine (TEA, >99%), 2-bromoisobutylyl bromide (BIBB, 98%), bis(2-dimethylaminoethyl) (methyl)amine (PMDETA, 99%), copper (I) bromide (99.999%), acrylamide (AAM, electrophoresis grade, >99%), 1-vinyl-2-pyrrolidone (NVP, >99%) and N-vinylcaprolactam (NVCL, 98%) were purchased from Sigma-Aldrich (St. Louis, MO, USA).

### 2.2. Methods

Graphene oxide and the obtained derived materials were dried under vacuum before analyses and applications. For FTIR spectroscopic measurements, a Thermo Nicolet iS10 FTIR spectrometer with an ATR accessory (SMART iTX) (Thermo Scientific, Waltham, MA, USA) was used. The baseline correction and normalization of the obtained spectra were performed using Omnic v9.0 software (Thermo Scientific). Atomic Force Microscopy (AFM) images were captured using a Dimension Icon AFM (Bruker, Santa Barbara, CA, USA) working in the PeakForce Tapping (PFT) and QNM^®^ modes. Standard silicon cantilevers for measurements in air (nominal spring constant of 0.4 N/m) were used. Elemental analyses were realized using a Vario Micro Cube Elemental Microanalyzer (Elementar, Langenselbolt, Germany). Raman spectroscopy and imaging measurements were performed using a WITec alpha 300 Confocal Raman Imaging system (WITec GmbH, Ulm, Germany) equipped with a charge-coupled device (CCD) detector (DU401A-BV-352, Andor, Belfast, United Kingdom). The air-cooled solid-state laser with an excitation wavelength of 532 nm was used to excite the samples. Scanning electron microscopy (SEM) images were captured on an Apreo S LoVac SEM (Thermo Fisher Scientific, Waltham, MA, USA). The SE mode with an acceleration voltage of 2.0 kV was used for the measurements.

Mechanical properties of nanocomposites, such as yield point, yield point elongation, tensile strength, elongation at break, tensile modulus, flexural modulus, flexural strength, Charpy notched impact strength and Charpy impact strength, were analyzed by a Static Materials Testing Machines ZWICK Z010 and ZWICK 5102 universal impact hammer (Zwick, Ulm, Germany). The samples with standard dimensions (ISO 527-1, ISO 527-2) prepared by an injection molding machine were used for the analyses.

TC of the prepared nanocomposites was determined using the pulse-power method of the Thermal Transport Option of the Physical Property Measurement System (Quantum Design, San Diego, CA, USA). The samples in the form of cylinders (d = 6 mm, h = 3 to 6 mm) were formed by the injection molding process. Measurements were carried out for five samples of each nanocomposite, and the average values were determined. TC was measured under vacuum in a steady state at room temperature. Heat pulses were injected into the samples by a heater which was connected to a copper electrode attached to the sample surface.

### 2.3. Grafting of Polymer Brushes from GO Surface

#### 2.3.1. Modifications of GO Sheets with APTES

The first step of surface modification of GO was performed in a THF solution of APTES (10 vol%), serving as the coupling agent, resulting in the formation of GO grafted with APTES (GO_APTES). After vacuum drying of GO for 2 h, it was placed in the APTES solutions for 24 h at room temperature. The obtained GO_APTES was rinsed with THF several times and shaken to remove possible residuals of unbound APTES.

#### 2.3.2. Immobilization of Initiator on GO_APTES

The freshly obtained GO_APTES (c.a. 2.2 g) was dispersed in THF (200 mL) containing TEA (4 mL). Then, BIBB (3.7 mL) was added dropwise to the reaction flask under argon atmosphere. After 1 h at room temperature, the reaction was terminated and the resulting GO_BIBB was centrifuged and rinsed to remove unreacted reagents with THF and water.

#### 2.3.3. Grafting of Polymer Brushes from GO_BIBB

Polymer brushes were grafted from GO_BIBB via SI-ATRP. The reaction system consisted of three flasks sealed with septa. Each flask was connected with double-tipped needles to provide transfer of reagents under argon atmosphere. The first flask contained the mixture of methanol/water (80:20 *v*/*v*, 250 mL) with a given monomer (0.35 mol) and PMDETA ligand (4.5 mL). Magnetic stirring bar and CuBr (4.26 g) were placed in the second flask, while GO_BIBB was put into the last vial (with the stirring bar). To ensure an inert atmosphere in the reaction system, it was purged with argon for 30 min. The degassed solution with the ligand and monomer from the first flask was transferred via the needle to the second flask with catalyst using flow of argon. The obtained mixture was stirred for 30 min to dissolve CuBr. Afterward, the reagents were transferred to the last flask with GO_BIBB to start the ATRP polymerization. The mixture was left to react for 2 h at ambient temperature with stirring, and the samples were collected after 5 min, 20 min, 1 h and 2 h during the polymerization process if necessary. The resulting product was separated and purified from the residual reagents and free polymer chains by washing a few times with methanol and water. For the polymerization process, the following monomers were used: AAM, NVCL and NVP, and the following samples were prepared: GO_PAAM, GO_PNVCL and GO_PNVP. All the samples were characterized using elemental analysis, while for preparation of the composites and further studies, only the samples after 2 h of polymerization were used.

### 2.4. Preparation of PA6 Nanocomposites

Nanocomposites were prepared by a melt compounding process using a conical shape laboratory twin screw extruder REM-2CA VERTEX (Zamak Mercator, Skawina, Poland) and injection molded by IM-15 (Zamak Mercator). PA6 powders were dried at 80 °C in a vacuum for 8 h before compounding with modified graphene materials. The temperature profile of heated zones from feeding hooper to the die was set to 90, 200, 260, 270, 260 and 250 °C, respectively, with the constant screw rotation speed of 50 rpm. The melted compound was directly introduced to the heated transfer cylinder (260 °C) equipped with piston and injected into the form (90 °C). Nanocomposites were prepared with nanoadditives (GO and GO with polymer brushes: GO_PAAM, GO_PNVP and GO_PNVCL) contents of 1%. Standard samples for mechanical measurements were prepared according to ISO 527-1 (conditioning in 60% moisture for 48 h), while for TC measurements, appropriate cylindrical samples were formed.

## 3. Results

### 3.1. Preparation of GO_PAAM via SI-ATRP

The main goal of the presented studies was the fabrication of the PA6-based nanocomposite with improved thermal conductivity and mechanical properties. For that purpose, GO sheets were decorated with PAAM brushes to enable homogenous dispersion of such modified nanofillers and integration within the PA6 matrix. The PAAM brushes were selected as they should form intermolecular hydrogen bonds with PA6 chains reinforcing the formed nanocomposite.

**Figure 1:**
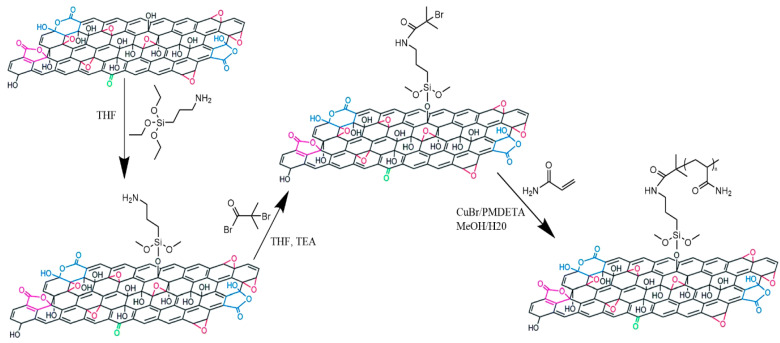
Synthetic route leading to formation of PAAM brushes on graphene oxide (GO): APTES grafting (first step), BIBB treatment (second step) and surface-initiated atom transfer radical polymerization (SI-ATRP) of acrylamide (third step).

In the first step, APTES molecules were chemisorbed on the GO surface since they serve as the anchoring points for the immobilization of the ATRP initiator molecules. APTES molecules containing terminal amine groups were attached via alkoxy groups to surface hydroxyl groups of GO. GO_APTES was reacted in the next step with BIBB in THF in the presence of TEA, leading to the formation of initiator-decorated GO_BIBB. The obtained GO_BIBB was then used for SI-ATRP of acrylamide. The applied synthetic route is shown schematically in Figure 1.

ATRP was selected as it enables controlled polymerization of the AAM monomer [26] required for homogenous decoration of the GO sheets with high enough grafting density to form polymer brushes with stretched conformation of the PAAM chains.

**Figure 2:**
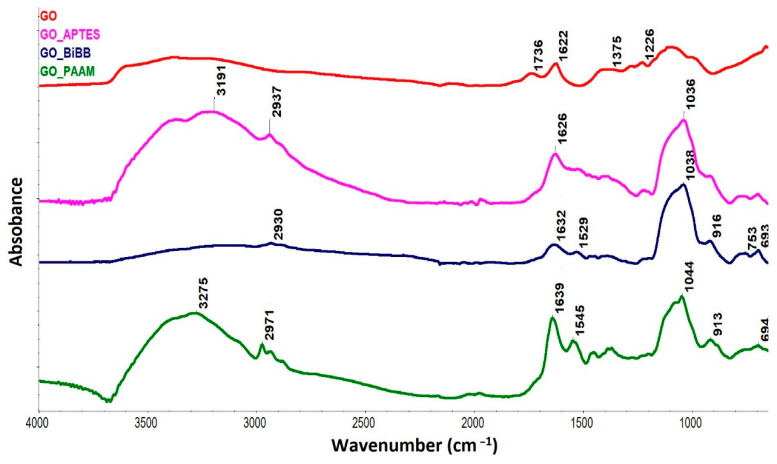
Normalized FTIR spectra of the pristine GO sheets and after subsequent modification with APTES (GO_APTES) and BIBB (GO_BIBB) and SI-ATRP of acrylamide (GO_PAAM).

Successful attachment of APTES to the GO surface was confirmed by FTIR analysis (Figure 2). The FTIR spectrum of GO contains a broad band in the region 3000–3700 cm^−1^, which can be assigned to the stretching vibrations of the O–H groups of the oxidized form of graphene [27]. The band at 1738 cm^−1^ in the GO spectrum can be assigned to the stretching vibrations of C=O in the carboxyl groups, which are present on the edges of GO or its structural defects [28]. The band at ca. 1400 cm^−1^ can be assigned to the deformation vibrations of the O–H groups [29]. The band range from 1500 to 900 cm^−1^ is most likely related to the vibrations of the C–O–C groups, while the bending C=C vibrations are responsible for the appearance of the band at 1622 cm^−1^ [30]. The IR spectrum of GO_APTES confirms the effectiveness of the reaction, due to the presence of the bands at about 2900 cm^−1^, which are responsible for the vibrations of the C–H bonds present in APTES. In addition to the bands characteristic of the C–O stretching of GO, the new strong bands appeared in the region of 1000–1200 cm^−1^, which can be assigned to the Si–O–C_graphene_ bonds [31], indicating covalent attachment of APTES molecules to the GO surface. Importantly, the intensity of the IR band in the range 3000–3700 cm^−1^ significantly increased, which can be attributed to the increased contribution of N–H stretching related to the presence of –NH_2_ groups of APTES but also the possible presence of residual water (–OH vibrations, hydrogen bonds). Comparing the FTIR spectra of GO_APTES and GO_BIBB, the changes may be noticed, especially in the region corresponding to the presence of amide bonds (1529 cm^−1^), which may be formed between APTES and BIBB. Moreover, the N–H vibration mode appearing at 1626 cm^−1^ for GO_APTES was shifted to 1632 cm^−1^ in GO_BIBB, indicating the attachment of BIBB [32]. However, the larger intensity of this broader band for GO_APTES may also be attributed to the presence of residual water (also adsorbed from air), as it was previously observed for APTES layers [33] and correlates here with the strong band in the 3000–3600 cm^−1^ range.

The obtained GO_BIBB was then used to perform SI-ATRP of acrylamide (Figure 1, step 3). The grafting of PAAM was followed by AFM, FTIR and elemental analysis. Successful grafting of PAAM brushes on the GO was evidenced by the appearance of a peak at 1545 cm^−1^, assigned to the deformation vibration of N–H in PAAM and at 1639 cm^−1^ corresponding to the stretch vibration of amide bonds, as well as the peak at 2971 cm^−1^ corresponding to the C–H stretching of main chain [34]. Moreover, the intensity of the band in the range of 3200–3400 cm^−1^ was significantly enhanced due to the presence of amide side groups in PAAM chains (the bands attributed to N–H stretching vibrations overlap with the bands assigned to O–H stretching vibrations).

**Figure 3:**
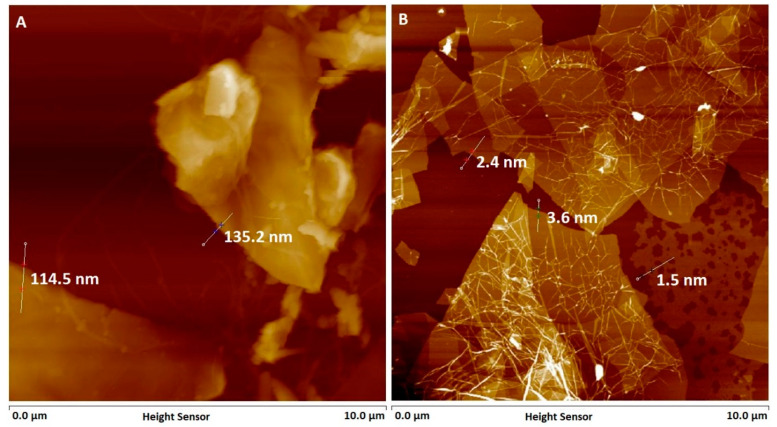
AFM topography images in air of GO_PAAM (**A**) and the native GO (**B**) with marked selected heights of the objects.

Figure 3 shows AFM images of the sample of GO and GO decorated with the PAAM brushes. The heights of the representative samples are marked in the pictures. The image of the native GO (Figure 3B) clearly shows the flat, 2D morphology of the material. For GO, the sample height was found to be 1.5 nm, which is greater than the theoretical value of one graphene layer. This difference is the result of the graphene oxidation process, which introduces oxidized groups onto the surface of the material and changes the hybridization of carbon atoms. It is also clear that GO flakes overlap each other and have various thicknesses, as seen in the AFM image (Figure 3B). Structurally, the material is not homogeneous, which is the result of the production process involving oxidation of graphite, intercalation of graphene flakes with oxidized forms and exfoliation as a result of ultrasound treatment. For some flakes, numerous wrinkles were observed, which is the result of capillary force acting on the GO surface during drying as reported previously [35]. The image of GO_PAAM (Figure 3A) still reveals flat structures but with much larger thicknesses (114.5 and 135.2 nm as indicated in the image), which are associated with the presence of polymer chains on the surface of graphene oxide. Polymer brushes grew on two sides of the surface of GO; therefore, it can be stated that the thickness of the dry PAAM chains is equal to about 60 nm. The bends and wrinkles visible in the AFM image of GO are not visible in the GO_PAAM image due to the presence of a relatively thick layer of PAAM. Elemental analysis confirmed the effectiveness of grafting of the polymer brushes (Table S1 in supplementary materials file). The progress of the polymerization was followed by measuring the nitrogen content, which is proportional to the mass of the grown PAAM brushes. Judging from the plot of the nitrogen content versus the polymerization time (Figure S1 in supplementary materials), one may conclude that the SI-ATRP proceeds regularly up to ca. 2 h when the polymer growth reaches a plateau. Such a behavior may be explained by crowding of the growing macroradicals for longer chains leading to enhanced termination of the radicals [36,37]. Based on the results of the elemental analysis (N:C ratio), the content of PAAM brushes in GO_PAAM after 2 h of polymerization was estimated to be ca. 31%.

Similarly, polymer brushes based on poly(1-vinyl-2-pyrrolidone) (PNVP) and poly(N-vinylcaprolactam) (PNVCL) were formed. Based on the results obtained for grafting PAAM brushes, the polymerization time was limited to 2 h for all the systems. The growth of the respective polymer brushes was confirmed by the observation of characteristic bands in the FTIR spectra (Figure S2 in supplementary materials). For example, the carbonyl band was observed at 1665 cm^−1^ in the spectrum of PNVCL brushes [38], whereas in the spectrum of PNVP, it appeared at 1655 cm^−1^ [39]. A change in the topography and thickness of the object with respect to the native GO samples was visualized using AFM (Figure S3 in supplementary materials). The content of the polymers in the decorated GO was found to be 23% for both GO_PNVCL and GO_PNVP, based on the elemental analysis results (Table S1 in supplementary materials).

### 3.2. Preparation and Characterization of PA6-Based Nanocomposites

The nanocomposites containing the same percentage, 1%, of the fillers (GO, GO_PAAM, GO_PNVCL and GO_PNVP, respectively) were prepared by extrusion followed by injection molding. The properties of the formed nanocomposites with decorated GO were compared to the one containing the parent GO and related to the properties of the PA6 matrix. Due to the contribution of the tethered polymer brushes, the actual content of GO in the PA6/GO_PAAM was even smaller than in PA6/GO and equal to ca. 0.69% of GO. Similarly, for PA6/GO_PNVCL and PA6/GO_PNVP, the net content of GO was equal to 0.77%.

**Figure 4:**
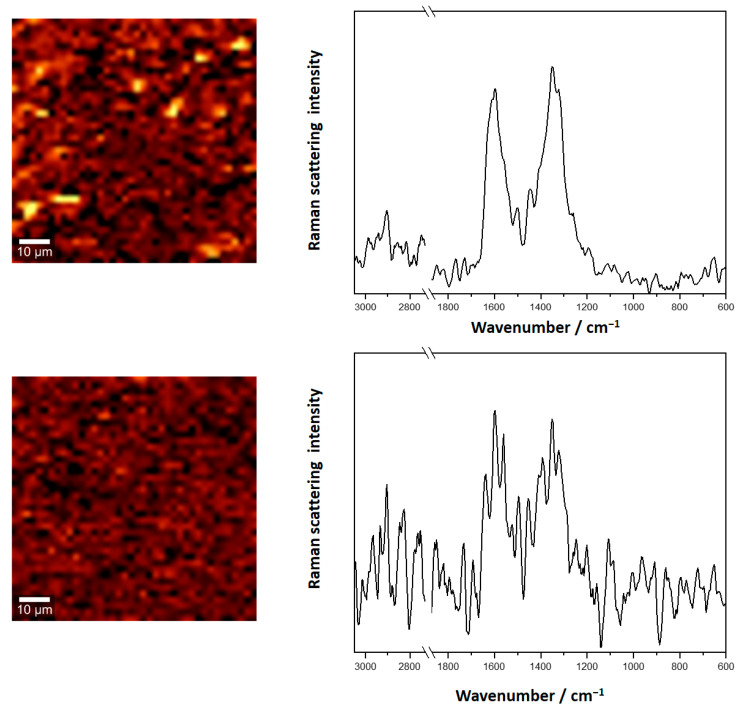
Raman images (integration of D band at 1350 cm^−1^; sampling density—2 μm) of PA6/GO_PAAM (top) and PA6/GO (bottom) nanocomposites together with respective Raman spectra.

**Figure 5:**
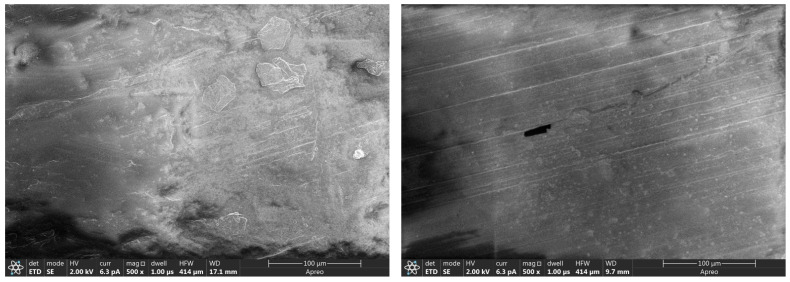
SEM images of PA6/GO (**left**) and PA6/GO_PAAM (**right**) nanocomposites.

The presence and distribution of the fillers in the nanocomposites was followed using Raman imaging (Figure 4) and SEM (Figure 5). The Raman images (integration of D-band at 1350 cm^−1^ characteristic of GO) revealed relatively homogenous distribution of GO in PA6/GO_PAAM with some distinct locations with higher signal intensity (the actual size of those spots may be overestimated due to the limitations of the planar resolution). On the contrary, only a very weak overall signal was observed for the nanocomposite containing native GO. In fact, intensity of the Raman bands in the spectrum of PA6/GO_PAAM is much higher than that observed for PA6/GO, for which the band intensity is only slightly higher than the noise level, in spite of a similar content of GO for both samples. This can be explained by the formation of clusters of GO in PA6/GO, which leads to a decrease in the band intensity, as observed previously [40,41,42].

Similarly, the SEM image of PA6/GO (Figure 5, left) shows large objects that can be assigned to the aggregates of GO, while decorated GO_PAAM seems to be uniformly distributed in the PA6 matrix (small light spots, Figure 5, right).

#### 3.2.1. Mechanical Properties

Mechanical properties of PA6/GO_PAAM and PA6/GO nanocomposites and the parent PA6 are shown in Table 1, and the representative curves are shown in Figure S4 in supplementary materials.

Tensile forces acting on the polymer material in the initial phase reveal its elastic properties. Then, after reaching the yield point, irreversible changes in the shape of the samples occur until they break. The addition of unmodified GO did not affect the tested mechanical properties of the composite. This may indicate a rather nonhomogeneous distribution of the nanoadditive and the lack of interactions between the filler and the matrix polymer. In the case of the PA6/GO_PAAM in comparison to PA6/GO and PA6, a clear difference can be observed for the yield point elongation value that increased from 11 to 24% (increase by 118%). The presence of polymer brushes on the GO_PAAM surface seems to promote stronger interactions between the matrix polyamide and surface-tethered poly(acrylamide) chains. The intermolecular hydrogen bonds can be formed at the interface, integrating the filler platelets with the matrix and reducing the possibility of breaking the polymer chains during stretching. In addition, the large surface area of the nanoadditive reduces the number of entanglements in the polymer matrix that significantly limit the translations of the polymer matrix chains. Additionally, the tensile strength of the PA6/GO_PAAM nanocomposite was also found to be ca. 12% higher than the one found for PA6, while the other parameters were practically not affected by the addition of the GO decorated with PAAM polymer brushes. Specifically, the elongation at break remained unchanged, while typically for such nanocomposites, it is significantly reduced due to possible aggregation of the nanofillers [43]. The mechanical parameters of PA6/GO_PNVCL and PA6/GO_PNVP indicate a rather worse integration of the fillers with the matrix, as the yield point elongation as well as elongation at break decreased significantly with respect to the pristine PA6 and PA6/GO_PAAM (Table 1 and Table S2 in supplementary materials).

Due to the fact that the addition of carbon materials often reduces the impact strength of composites, a Charpy impact test was performed [44]. Importantly. the analyzed PA6/GO_PAAM samples were not damaged during the notched impact analysis (unlike the nanocomposites with the other decorated GO), while notched samples did not differ much in their impact values.

#### 3.2.2. Thermal Conductivity

**Figure 6:**
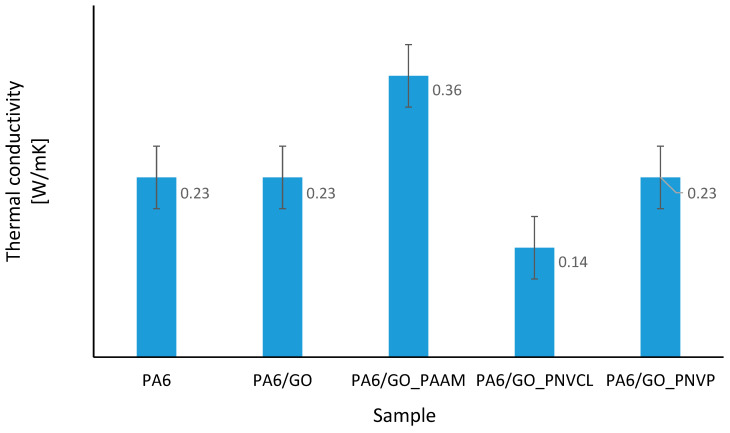
Thermal conductivity of native PA6 and respective nanocomposite samples.

Thermal conductivity values of the prepared nanocomposite samples are shown in Figure 6. The value of thermal conductivity of PA6 was found to increase by 54%, reaching 0.36 W/mK, with the introduction of only 1% of PA6/GO_PAAM into the polymer matrix. Heat transport in polymeric materials occurred due to phonons, which propagate through the network; therefore, good thermal conductivity requires a strong filler-polymer interface [45].

**Scheme 1:**
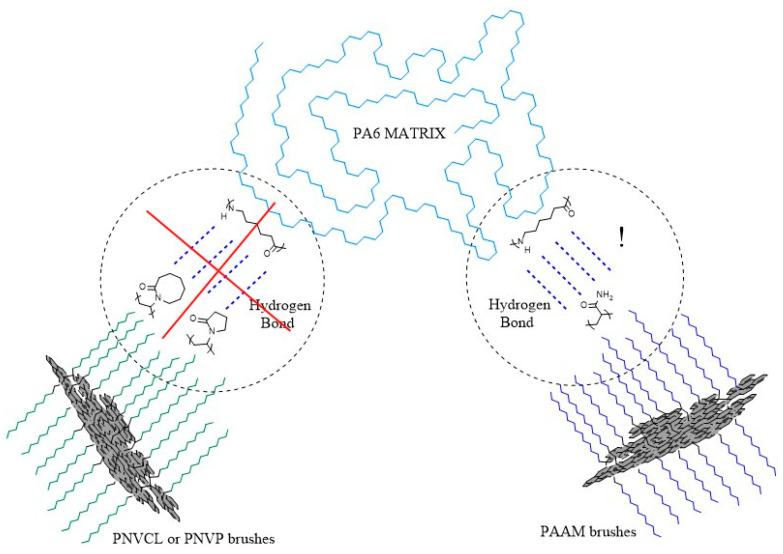
Presentation of the role of hydrogen bonds in strengthening of interfacial interactions between the polymer brushes decorating GO fillers and the macromolecules of the PA6 matrix.

The presence of polymer brushes in the PA6/GO_PAAM nanocomposite clearly changes the interfacial properties. PAAM chains covalently attached to GO are able to reduce the phonon scattering thanks to their interaction with the polymer matrix chains. The coiled chains attached at low grafting density to the surface of GO might not interpenetrate the matrix chains, thus limiting the compatibilizing effect. For samples containing pristine GO or decorated with other polymer brushes (PNVCL and PNVP), thermal conductivity values are similar or even lower in comparison with PA6, despite the even higher net content of GO in comparison to PA6/GO_PAAM. In the case of PNVP and PNVCL, hydrogen bonds are mainly intermolecular, leading to exposure of hydrocarbon rings to contact with the PA6 matrix [38,46]. These phenomena are responsible for the lack of hydrogen bonds at the interface with PA6 chains and result in a less efficient interaction between the GO-grafted polymer brushes and the PA6 matrix chains than in the case of PAAM (see Scheme 1). Thus, lower thermal conductivity was observed for the nanocomposites containing GO_PNVCL and GO_PNVP in comparison to PA6/GO_PAAM, for which intermolecular hydrogen bonds may be formed at the interface that make the phonons propagation more effective. Unfortunately, due to the very low content of the fillers and the overlaying of the indicative bands with other bands in the systems, the formation of the mentioned hydrogen bonds could not be revealed in the FTIR spectra of the nanocomposites.

## 4. Conclusions

Graphene oxide flakes, decorated with polyacrylamide brushes obtained in the surface-initiated ATRP, were used to form nanocomposites with a model polyamide (PA6), leading to significantly enhanced (by 54%) thermal conductivity and mechanical properties without affecting ductility. ATRP coupling agent was used to introduce amine groups on the graphene oxide surface that in the next step were used to covalently attach the ATRP initiator groups. The surface-initiated polymerization led to the formation of PAAM polymer brushes of thicknesses up to ca. 60 nm on the graphene oxide surface, as determined by elemental analysis, AFM imaging and FTIR spectroscopy.

To assess the impact of the developed hybrid material on the mechanical properties and thermal conductivity, composite samples of PA6. PA6/GO and PA6/GO_PAAM were prepared by extrusion and followed by injection molding. The addition of graphene oxide reduces the number of entanglements of the polymer matrix and prevents the isotropic transfer of forces imposed on the sample, which results in the deterioration of strength properties. Graphene oxide with polyacrylamide brushes introduces additional functional groups at the polymer-filler that can form hydrogen bonds with polymer matrix chains. As indicated by SEM and Raman imaging, GO fillers decorated with PAAM brushes were homogenously distributed in the PA6 matrix, unlike native GO, which seemed to form aggregates in the composite. Owing to this, for the PA6/GO_PAAM sample, the elastic deformation range was increased, and the elongation at break value was not affected.

This work was intentionally limited to the impact of nanofiller in the amount of 1%, which seems economically feasible since graphene-based materials are relatively expensive, limiting their broad applicability as additives for plastics. The introduction of only 1% of graphene oxide with polyacrylamide brushes improved the thermal conductivity of the composite by 54% compared to the raw PA6, preserving good impact strength. The presence of dense polymer brushes grafted from 2D nanofillers enhanced interactions between the phases, thus reducing dispersion at the interface and facilitating phonon transport. The presented approach may pave the way for further development of nanocomposites with a low content of nanofillers, which brings desirable properties (e.g., enhanced thermal conductivity) with no negative effects on the valuable properties of the polymer matrix that are otherwise commonly affected at a high content of nanofillers.

## Figures and Tables

**Table 1:** Mechanical properties of the PA6/GO_PAAM and PA6/GO nanocomposites and parent PA6. (Numbers in bold indicate significant differences).

Properties	Unit	PA6/GO_PAAM	PA6/GO	PA6
Yield point	MPa	40.0 ± 0.9	42.0 ± 1.2	41.0 ± 1.2
Yield point elongation	%	**24.0 ± 1.6**	11.0 ± 1.3	11.0 ± 1.2
Tensile strength	MPa	**38 ± 1**	34 ± 0.6	33.5 ± 0.6
Elongation at break	%	241 ± 14	240 ± 9	240 ± 9
Tensile modulus	MPa	820 ± 90	980 ± 100	968 ± 100
Charpy notched impact strength	kJ/m^2^	5.2 ± 0.1	5.0 ± 0.3	5.0 ± 0.3
Charpy impact strength	kJ/m^2^	no break	no break	no break

## Data Availability

The data is contained within the article and supplementary materials.

## Supplementary Materials

The following are available online at https://www.mdpi.com/1996-1944/14/4/751/s1, Figure S1: Plot of the PAAM content in the GO_PAAM samples versus the polymerization time, Figure S2: Normalized FTIR spectra of the GO_PNVP and GO_PNVCL, Figure S3: AFM topography images in air of (A) GO_PNVCL and (B) GO_PNVP with example heights of the samples, Table S1: Results of the elemental analyses of the parent GO and the GO samples decorated with polymer brushes together with the calculated polymer content, Table S2: Mechanical properties of the PA6/GO_PNVCL and PA6/GO_PNVP composites containing 1% of the decorated GO samples, Figure S4. Representative stress-strain curves obtained for PA6 and respective nanocomposites.
