# Supplementary material for: Enhanced Thermal Conductivity of Polyamide-Based Nanocomposites Containing Graphene Oxide Sheets Decorated with Compatible Polymer Brushes
Source: Materials (Basel). 2021 Feb 5;14(4):751. doi: 10.3390/ma14040751 (PMC7914667; doi:10.3390/ma14040751)
Supplement: Supplementary file 1 [file materials-14-00751-s001.zip › materials-1062926-supplementary.pdf]

materials

MDPI

Supplementary

Enhanced Thermal Conductivity of Polyamide-Based Nanocomposites Containing Graphene Oxide Sheets Decorated with Compatible Polymer Brushes

Łukasz Łątka [1,2], Kamil Goc [3], Czesław Kapusta [3] and Szczepan Zapotoczny [1,*]

[1] Faculty of Chemistry, Jagiellonian University, Gronostajowa 2, 30-387 Krakow, Poland; lat.lukasz@gmail.com
[2] Research and Development Center, Azoty Group S.A., Kwiatkowskiego 8, 33-101 Tarnow, Poland
[3] Faculty of Physics and Applied Computer Science, AGH University of Science and Technology, Mickiewicza Av. 30, 30-059 Krakow, Poland; kamil.goc@fis.agh.edu.pl (K.G.); kapusta@agh.edu.pl (C.K.)
* Correspondence: zapotocz@chemia.uj.edu.pl; Tel.: +48 12 686 2530

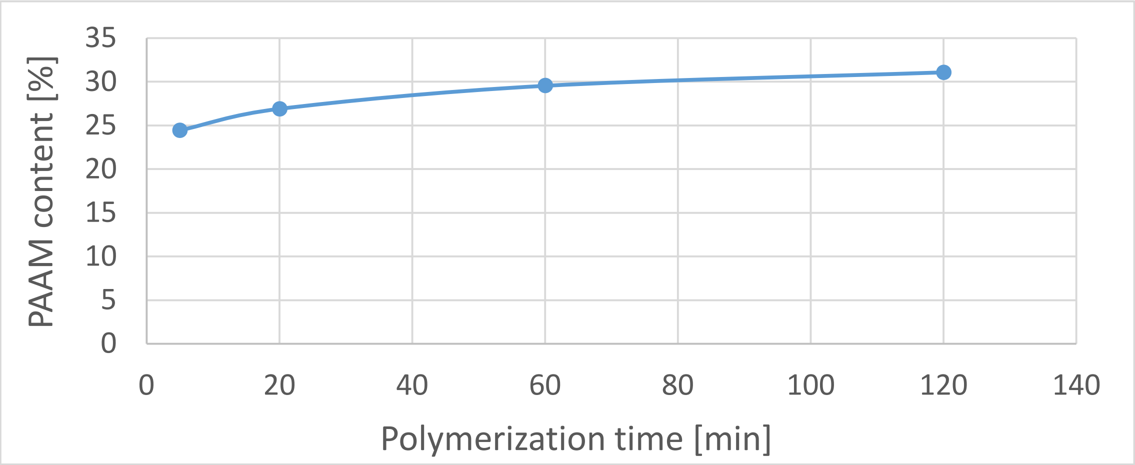

Figure S1. Plot of the PAAM content in the GO–PAAM samples versus the polymerization time.

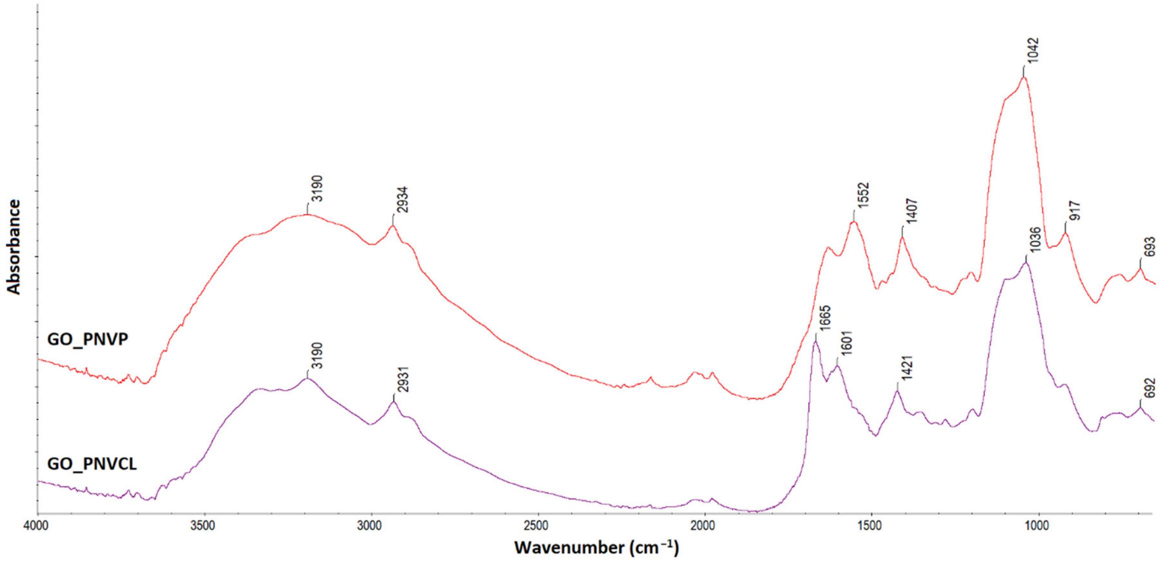

Figure S2. Normalized FTIR spectra of the GO_PNVP and GO_PNVCL.

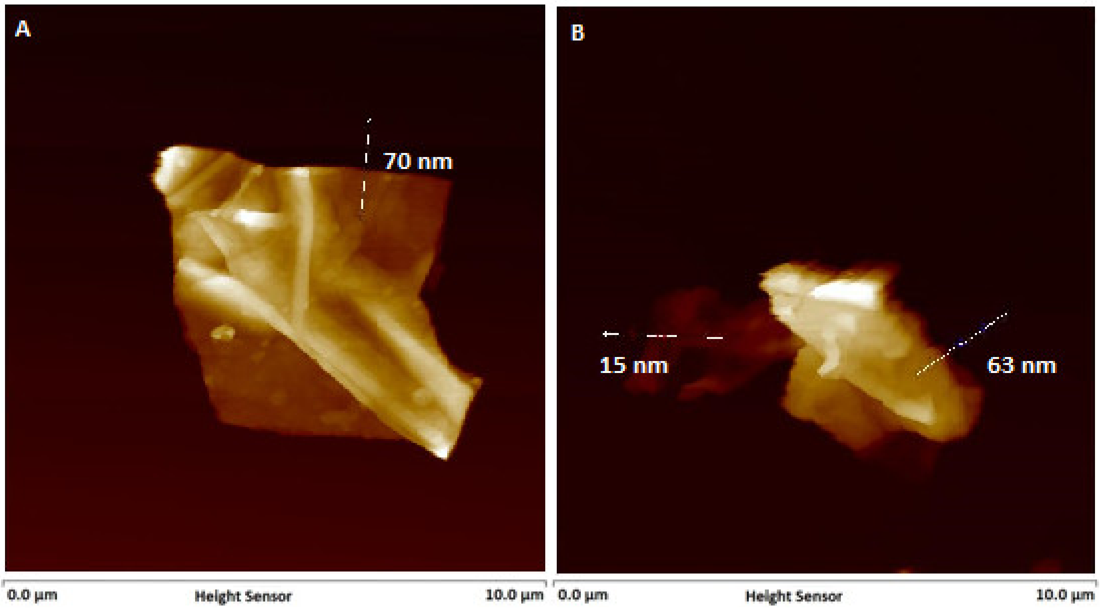

Figure S3. AFM topography images in air of (**A**) GO_PNVCL, and (**B**) GO_PNVP with example heights of the samples.

Table S1. Results of the elemental analyses of the parent GO and the GO samples decorated with polymer brushes together with the calculated polymer content.

Samples	N [%]	C [%]	H [%]	Polymer Content [%] based on C:N Ratio
GO	0	39.52	4.53	0
GO_PAAM 5 min	4.77	41.82	4.67	24
GO_PAAM 20 min	5.22	41.84	4.72	27
GO_PAAM 1 h	5.65	41.51	4.87	30
GO_PAAM 2 h	5.95	41.74	5.01	31
GO_PNVCL	4.83	44.90	5.00	23
GO_PNVP	4.70	44.54	4.84	23

Table S2. Mechanical properties of the PA6/GO_PNVCL and PA6/GO_PNVP composites containing 1% of the decorated GO samples.

Properties	Unit	PA6/GO_PNVCL	PA6/GO_PNVP
Yield point	MPa	47.6 ± 0.6	49 ± 0.9
Yield point elongation	%	6.4 ± 2.1	8.3 ± 1.1
Tensile strength	MPa	38.5 ± 1.1	42 ± 1.4
Elongation at break	%	161 ± 51	224 ± 13
Tensile modulus	MPa	1430 ± 32	1330 ± 51
Charpy notched impact strength	kJ/m^2	4.1 ± 0.1	4.0 ± 0.3
Charpy impact strength	kJ/m^2	49 ± 10	77 ± 25

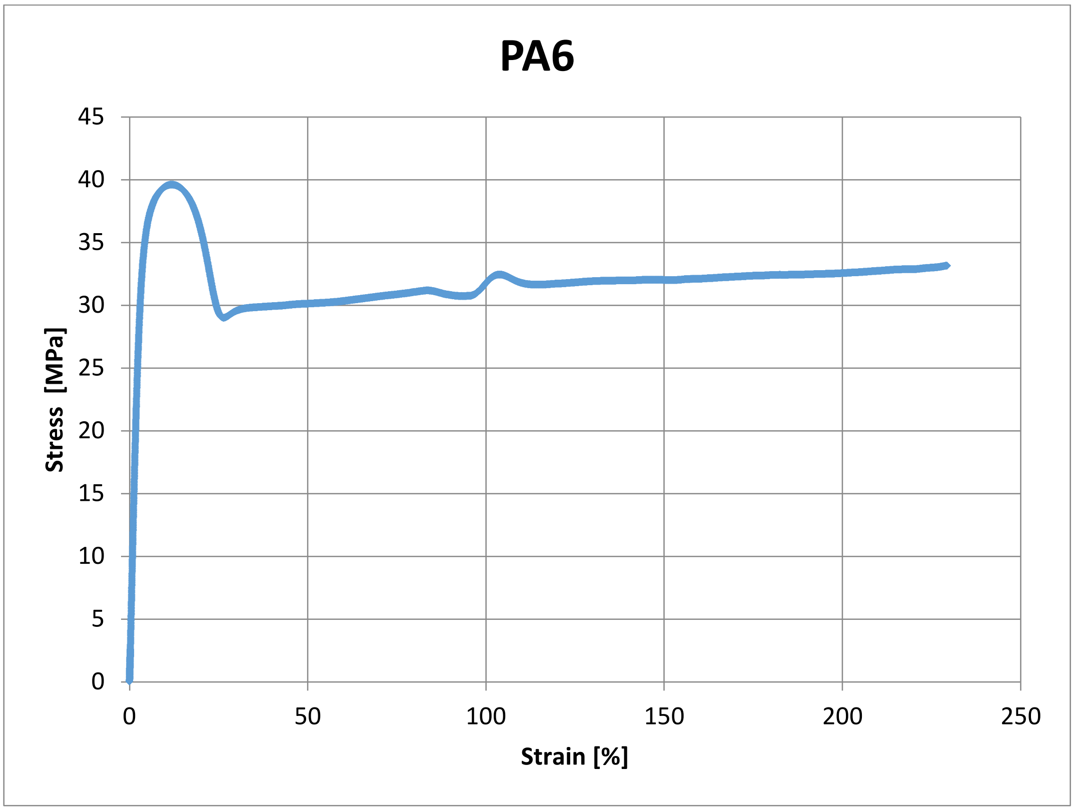
PA6
45
40
35
30
25
20
15
10
5
0
Stress [MPa]
0
50
100
150
200
250
Strain [%]

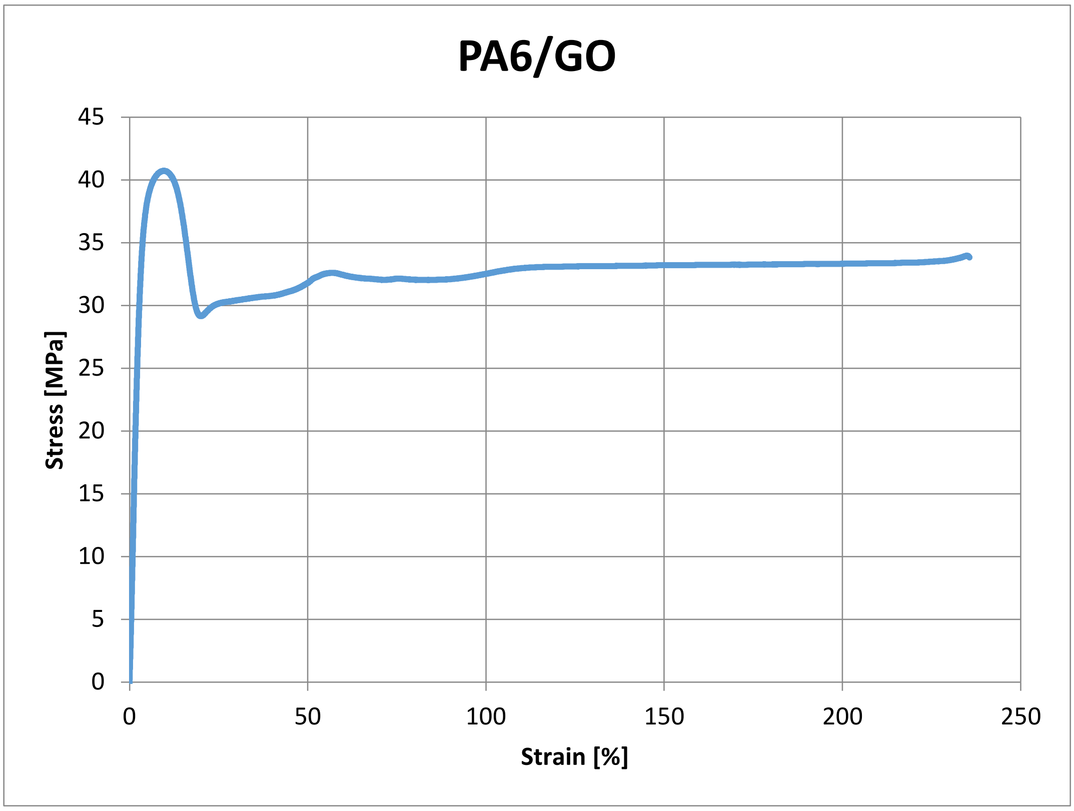
PA6/GO
45
40
35
30
25
20
15
10
5
0
Stress [MPa]
0
50
100
150
200
250
Strain [%]

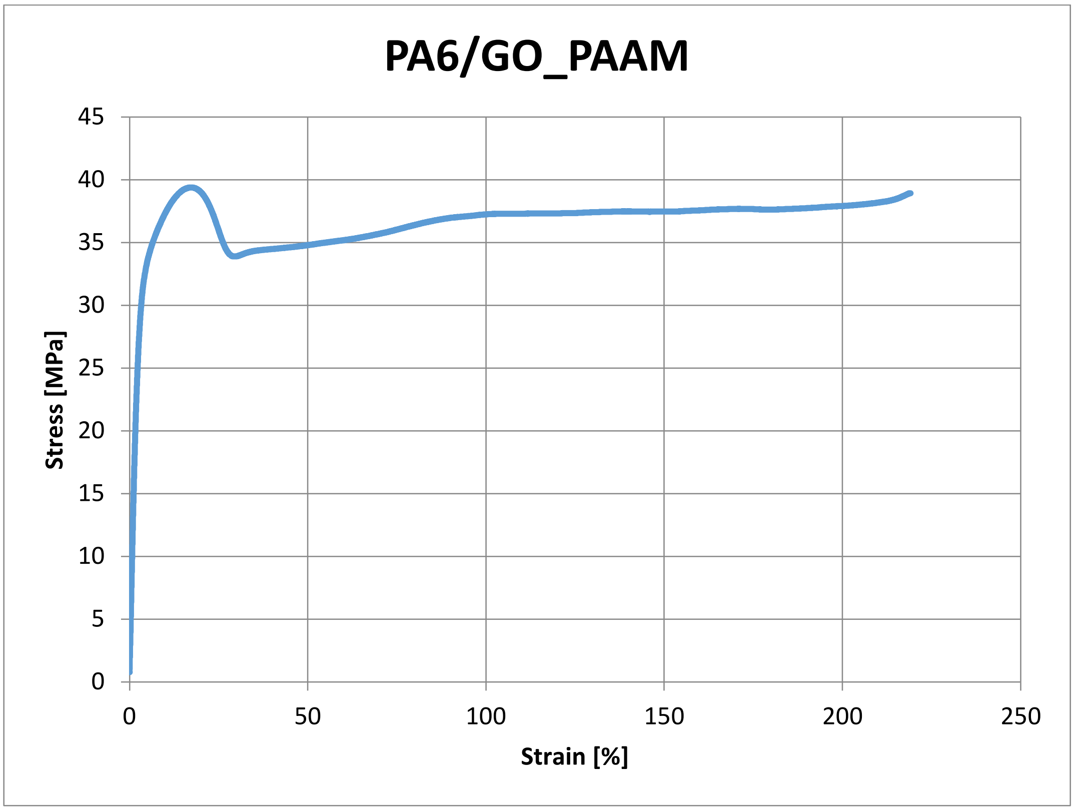
PA6/GO_PAAM
Stress [MPa]
0
5
10
15
20
25
30
35
40
45
0
50
100
150
200
250
Strain [%]

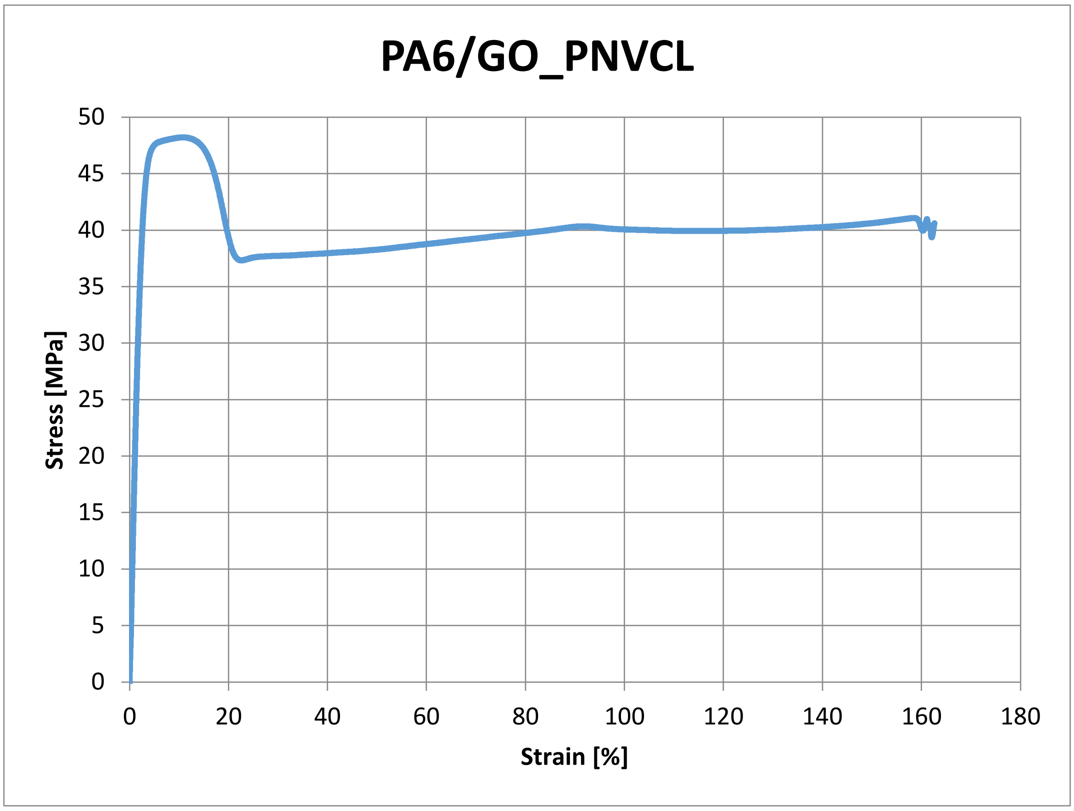
PA6/GO_PNVCL
Stress [MPa]
0
5
10
15
20
25
30
35
40
45
50
0
20
40
60
80
100
120
140
160
180
Strain [%]

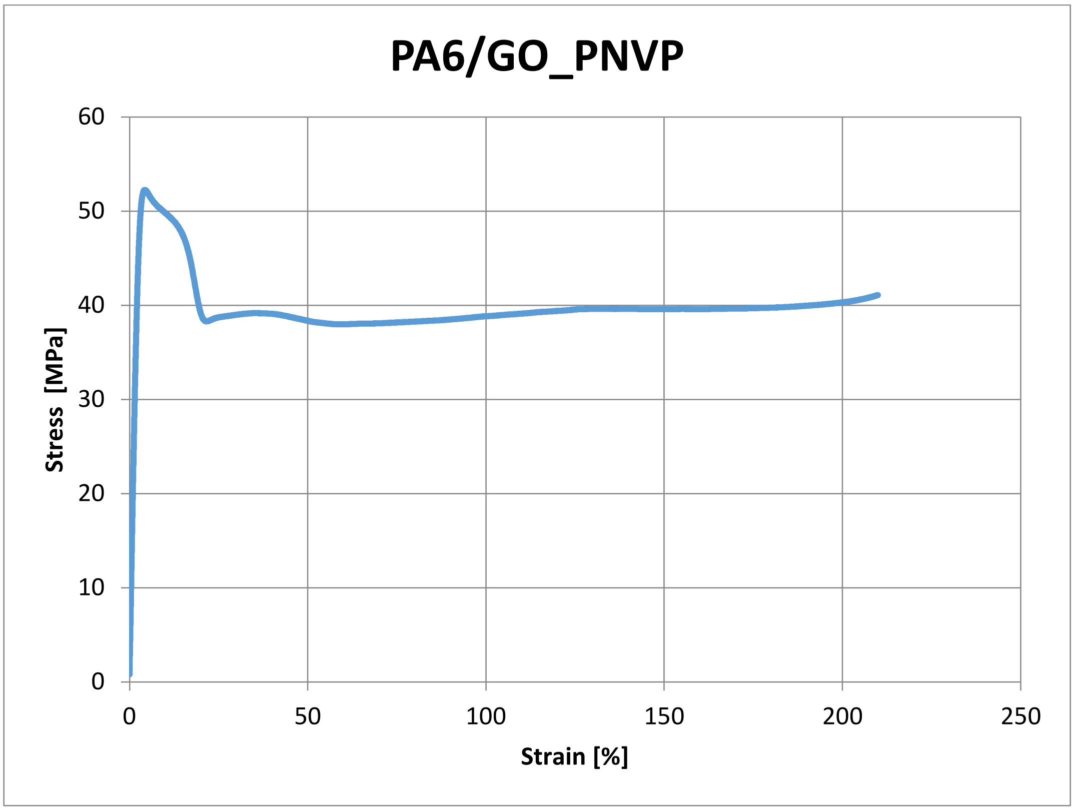

Figure S4. Representative stress-strain curves obtained for PA6 and respective nanocomposites.
